# Water in Food

**Published:** 1886-12

**Authors:** 


### WATER IN FOOD.

The action of water in our food, says a medical writer, is very important. There would be no carrying of food into the system but for the agency of water. It dissolves everything that we take, and nothing that we take as food can become nutriment that is not dissolved in water. It would not do to test that by taking things and putting them into water and seeing whether they dissolve, and rejecting them as food according to that circumstance ; because food undergoes a considerable change in the stomach. It undergoes a change, to begin with, in our mouth. One of the great objects of that change is to render things soluble which have been before insoluble in water. Starch, which we cannot dissolve in water out of the stomach, is dissolved in water directly it gets into the mouth, for the starch is changed by the saliva into sugar, and that which would lie unchanged in water for months is so changed by the saliva of the mouth and the gastric juice of the stomach that it is speedily dissolved. Hence, when we are taking considerable quantities of dry food, it becomes absolutely necessary that we should add a certain quantity of water, so that this dry food should become dissolved. Such things as oats, barley, wheat, rice, maize and other articles of diet containing little water, must have water added, in order that their starch, fat and gluten may be dissolved and enter into the system.

